# Supplementary material for: Loss of Notch dimerization perturbs intestinal homeostasis by a mechanism involving HDAC activity
Source: PLoS Genet. 2024 Dec 12;20(12):e1011486. doi: 10.1371/journal.pgen.1011486 (PMC11670933; doi:10.1371/journal.pgen.1011486)
Supplement: S1 Fig — (A) qPCR of gene expression of Lgr5 on RNA extracted from distal colon of N1+/+; N2+/+ and N1RA/RA; N2RA/RA mice treated for 10 days 1% DSS relative to controls, n = 3 mice per group. *p <0.05, **p <0.01, ***p <0.001. (B) Daily weight measurements of N1+/+; N2+/+; Lgr5-EGFP-IRES-creERT2 or N1RA/RA; N2RA/RA; Lgr5-EGFP- IRES-creERT2 mice treated with 2% DSS 5 days, all N1RA/RA; N2RA/RA; Lgr5-EGFP-IRES-creERT2 mice had to be euthanized within 6 days due to severe colitis-induced weight loss. *p <0.05, **p <0.01, ***p <0.001. (C) Immunofluorescence staining of GFP and Cytokeratin 8+18 in distal colon. White dashed box indicated zoomed region. Scale bars = 50 μm. (D) Quantification of number of GFP-positive cells per crypt. n = 3 mice per group. *p <0.05, **p <0.01, ***p <0.001. (PDF) [file pgen.1011486.s001.pdf]

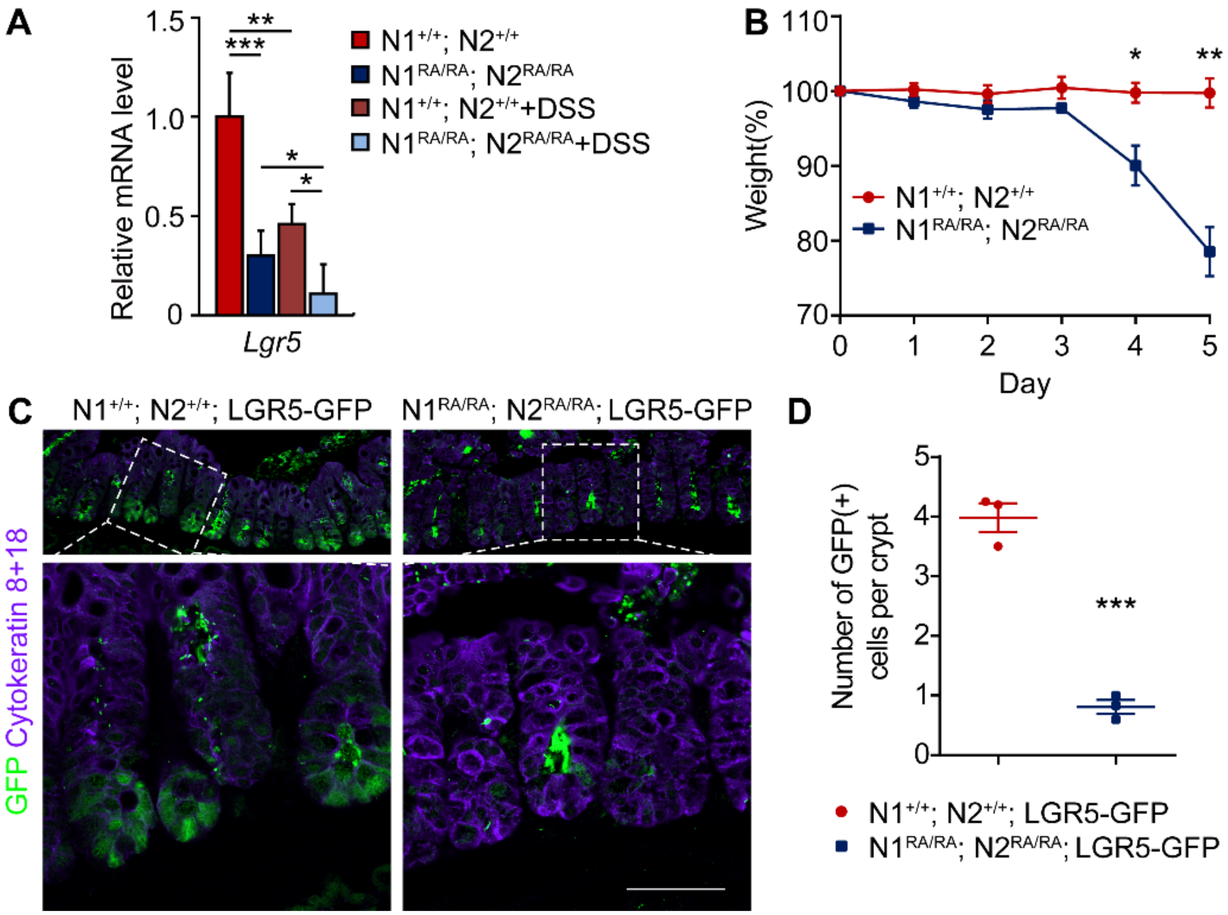

S1. Fig: NDD mice loose Lgr5^{+} stem cells in the colon.

A. qPCR of gene expression of *Lgr5* on RNA extracted from distal colon of $N1^{+/+}$; $N2^{+/+}$ and $N1^{RA/RA}$; $N2^{RA/RA}$ mice treated for 10 days 1% DSS relative to controls, n=3 mice per group. **p* <0.05, ***p* <0.01, ****p* <0.001.

B. Daily weight measurements of $N1^{+/+}$; $N2^{+/+}$; *Lgr5-EGFP-IRES-creERT2* or $N1^{RA/RA}$; $N2^{RA/RA}$; *Lgr5-EGFP-IRES-creERT2* mice treated with 2% DSS 5 days, all $N1^{RA/RA}$; $N2^{RA/RA}$; *Lgr5-EGFP-IRES-creERT2* mice had to be euthanized within 6 days due to severe colitis-induced weight loss. **p* <0.05, ***p* <0.01, ****p* <0.001.

C. Immunofluorescence staining of GFP and Cytokeratin 8+18 in distal colon. White dashed box indicated zoomed region. Scale bars=50 µm.

D. Quantification of number of GFP-positive cells per crypt. n=3 mice per group. **p* <0.05, ***p* <0.01, ****p* <0.001.
