# Supplementary material for: Loss of Notch dimerization perturbs intestinal homeostasis by a mechanism involving HDAC activity
Source: PLoS Genet. 2024 Dec 12;20(12):e1011486. doi: 10.1371/journal.pgen.1011486 (PMC11670933; doi:10.1371/journal.pgen.1011486)
Supplement: S2 Fig — Shown are all the biotinylated NICD2 peptides recovered by Streptavidin precipitation using PyMol (https://pymol.org/2/). For docking purposes, the structures were aligned on ANK, such that the RAM domain of NICD2 is floating in space and not docking on RBPj. For NICD2, all lysine residues are shown as “sticks” and the recovered peptides are colored green or cyan to distinguish overlapping peptides. The PEST domain and C-terminus of NICD2 are hidden since modelled it clashed with ANK. (PDF) [file pgen.1011486.s002.pdf]

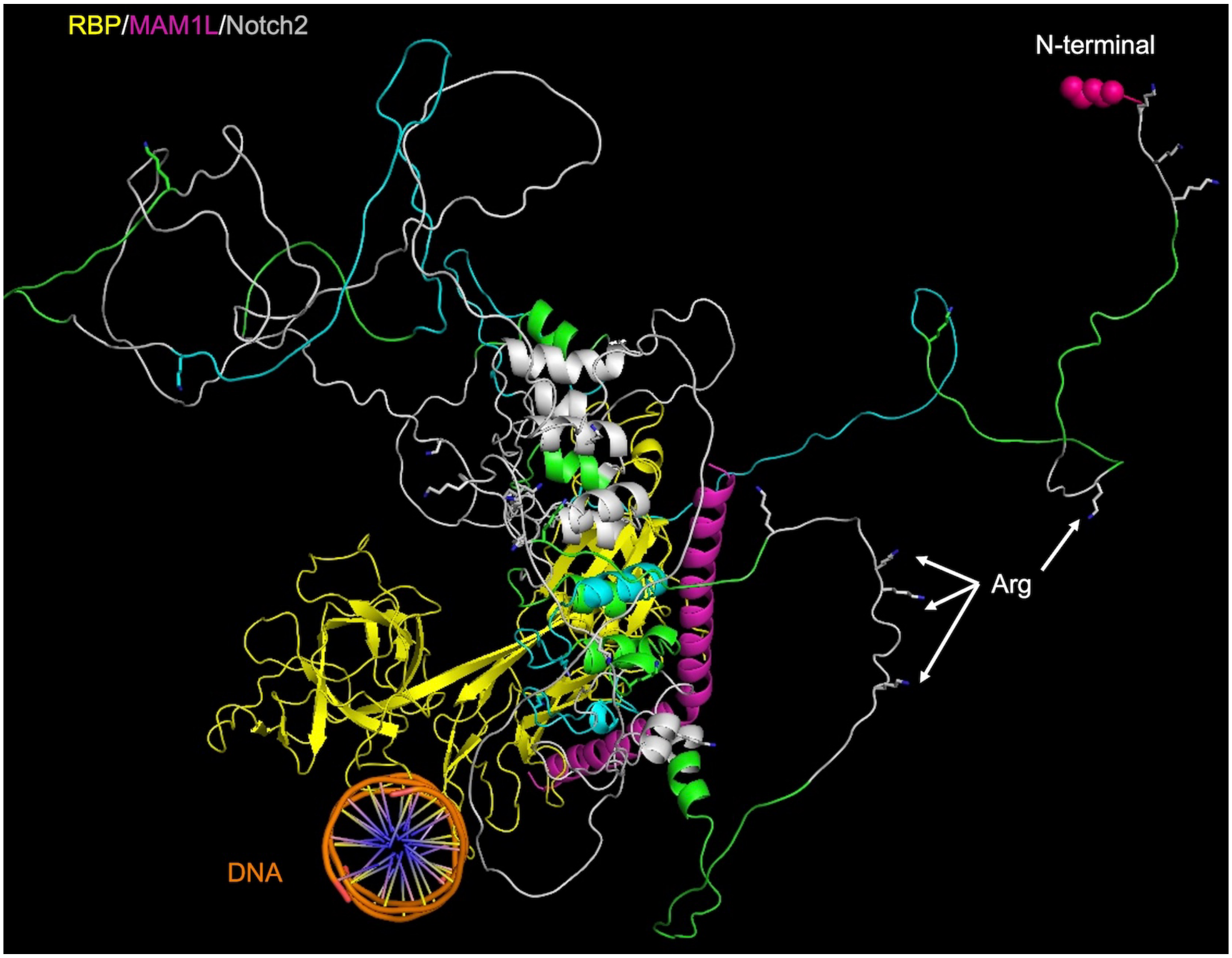


**S2. Fig: Mapping Biotinylated peptide onto NTC.**

Shown are all the biotinylated NICD2 peptides recovered by Streptavidin precipitation using PyMol (https://pymol.org/2/). For docking purposes, the structures were aligned on ANK, such that the RAM domain of NICD2 is floating in space and not docking on RBPj. For NICD2, all lysine residues are shown as “sticks” and the recovered peptides are colored green or cyan to distinguish overlapping peptides. The PEST domain and C-terminus of NICD2 are hidden since modelled it clashed with ANK.
