# Supplementary material for: Loss of Notch dimerization perturbs intestinal homeostasis by a mechanism involving HDAC activity
Source: PLoS Genet. 2024 Dec 12;20(12):e1011486. doi: 10.1371/journal.pgen.1011486 (PMC11670933; doi:10.1371/journal.pgen.1011486)
Supplement: S3 Fig — (A) Fold change of Lgr5, Ascl2, Prom1 gene expression in jejunum relative to wild type mice analyzed by qRT- PCR. n = 3 mice per group. Quantitative data are presented as mean ± SEM. ns-Not Significant (B-C) Immunofluorescence staining of Sox9 of jejunum (B) and colon (C). (D) Jejunum immunofluorescence staining of Cleaved Caspase-3. (E) Representative images of MMP7 (Paneth cell marker) and PCNA immunofluorescence in jejunum. (F-H) Representative jejunum immunofluorescence images of CgA (enteroendocrine cells, F), Dcamkl1 (tuft cells, G), and Muc2 (secretory cells, H). Scale bars = 100 μm. (PDF) [file pgen.1011486.s003.pdf]

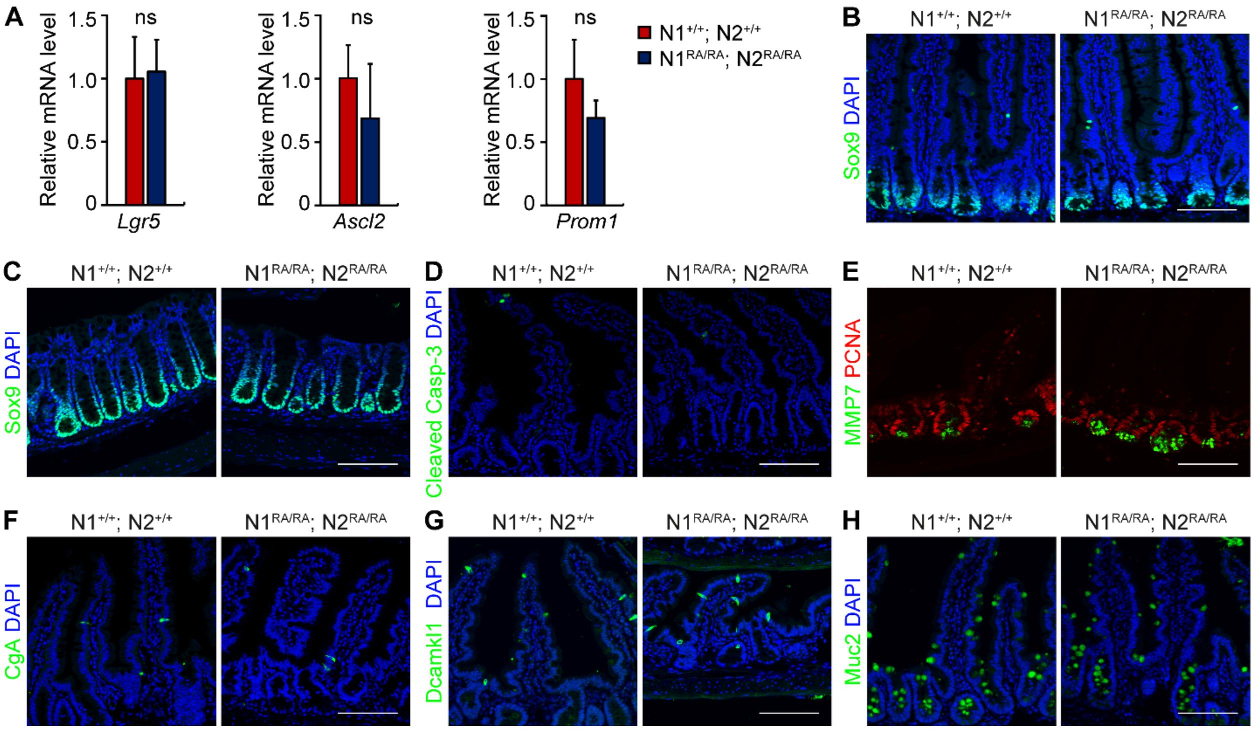

S3. Fig: Differentiation of intestinal cell types are not affected by Notch dimerization deficiency.

A. Fold change of *Lgr5*, *Ascl2*, *Prom1* gene expression in jejunum relative to wild type mice analyzed by qRT-PCR. n=3 mice per group. Quantitative data are presented as mean ± SEM. ns-Not Significant

B-C. Immunofluorescence staining of Sox9 of jejunum (B) and colon (C).

D. Jejunum immunofluorescence staining of Cleaved Caspase-3.

E. Representative images of MMP7 (Paneth cell marker) and PCNA immunofluorescence in jejunum.

F-H. Representative jejunum immunofluorescence images of CgA (enteroendocrine cells, F), Dcamkl1 (tuft cells, G), and Muc2 (secretory cells, H).

Scale bars=100 μm.
