# Supplementary material for: Loss of Notch dimerization perturbs intestinal homeostasis by a mechanism involving HDAC activity
Source: PLoS Genet. 2024 Dec 12;20(12):e1011486. doi: 10.1371/journal.pgen.1011486 (PMC11670933; doi:10.1371/journal.pgen.1011486)
Supplement: S4 Fig — (A-C) H&E staining of distal colon tissue from aged N1+/+; N2+/+, N1RA/RA; N2RA/RA and N1+/-; N2+/- mice. Scale bars = 100 μm. (A’-C’) Immunofluorescence staining of Ki67, and nuclei staining with DAPI in distal colon tissue from mice with the indicated genotypes. Scale bars = 100 μm. n = 3 mice per group. (PDF) [file pgen.1011486.s004.pdf]

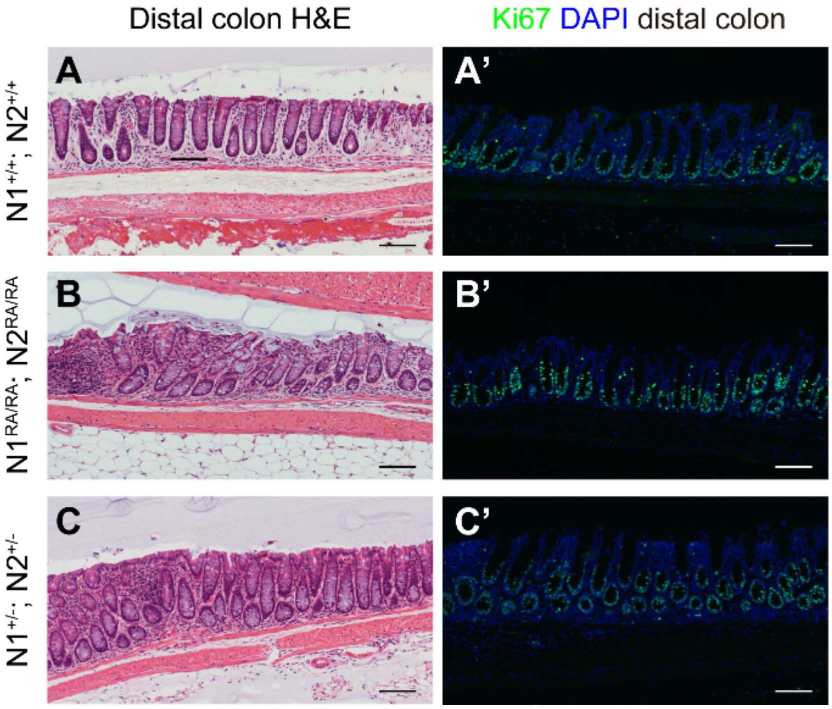

S4. Fig: Notch dimer-deficiency in mice does not impair colon homeostasis with age.
A-C. H&E staining of distal colon tissue from aged *N1*$^{+/+}$*; N2*$^{+/+}$, *N1*$^{RA/RA}$*; N2*$^{RA/RA}$ and *N1*$^{+/-}$*; N2*$^{+/-}$ mice. Scale bars=100 µm.
A'-C'. Immunofluorescence staining of Ki67, and nuclei staining with DAPI in distal colon tissue from mice with the indicated genotypes. Scale bars=100 µm. n=3 mice per group.
