# Supplementary material for: Loss of Notch dimerization perturbs intestinal homeostasis by a mechanism involving HDAC activity
Source: PLoS Genet. 2024 Dec 12;20(12):e1011486. doi: 10.1371/journal.pgen.1011486 (PMC11670933; doi:10.1371/journal.pgen.1011486)
Supplement: S5 Fig — (A) Co-IP analysis of isotype IgG control and Notch2 interaction with Hdac2. (B) Daily weight measurements of N1+/+; N2+/+ mice treated with DBZ (10 μmol/kg) or VPA (200 mg/kg/day) alone or DBZ plus VPA. n = 3 mice per group. ns-not significant. (PDF) [file pgen.1011486.s005.pdf]

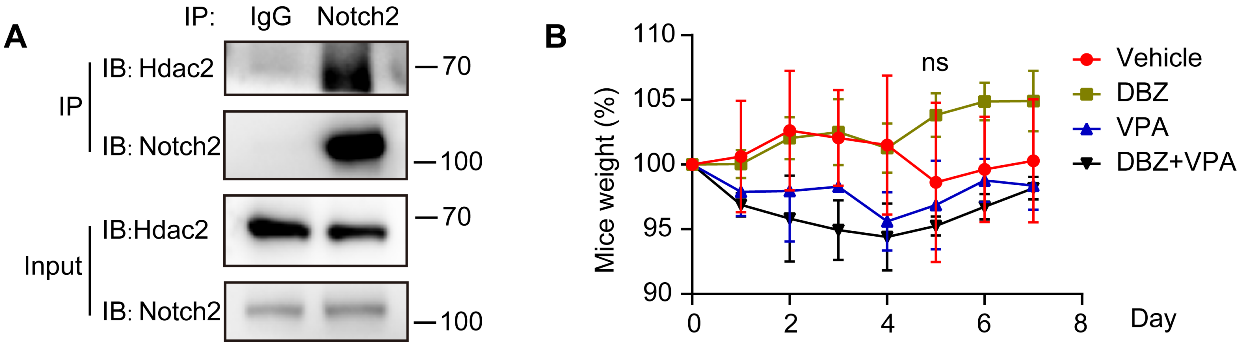

S5 Fig: Hdac2 interacts with Notch2. (A) Co-IP analysis of isotype IgG control and Notch2 interaction with Hdac2. (B) Daily weight measurements of *N1+/+; N2+/+* mice treated with DBZ (10 μmol/kg) or VPA (200 mg/kg/day) alone or DBZ plus VPA. n=3 mice per group. ns-not significant.
