# Supplementary material for: Loss of Notch dimerization perturbs intestinal homeostasis by a mechanism involving HDAC activity
Source: PLoS Genet. 2024 Dec 12;20(12):e1011486. doi: 10.1371/journal.pgen.1011486 (PMC11670933; doi:10.1371/journal.pgen.1011486)
Supplement: S6 Fig — The amino acid sequence of truncated human NICD2 and NICD2RA. The transmembrane domain is boxed in red dashed line. ARG(R1934) and ALA(A1934) are highlighted. Red arrow marks gamma secretase cleavage sites that will generate stable NICD2s. (PDF) [file pgen.1011486.s006.pdf]

NICD2 amino acid sequence:

SYPLVSVVSESLTPERTQLLYLLAVAVVIILFIILLGVIMAKRKRKHGSLWLPEGFTLRRDASNHKRRE
PVGQDAVGLKNLSVQVSEANLIGTGTSEHWVDDEGPQPKKVKAEDEALLSEEDDPIDRRPWTQQ
HLEAADIRRTPSLALTPPQAEQEVDVLDVNVRGPDGCTPLMLASLRGGSSDLSDEDEDAEDSSAN
IITDLVYQGASLQAQTDRTGEMALHLAARYSRADAAKRLLDAGADANAQDNMGRCPLHAAVAADA
QGVFQILIRNRVTDLDARMNDGTTPLILAARLAVEGMVAELINCQADVNAVDDHGKSALHWAAAVN
NVEATLLLLKNGANRDMQDNKEETPLFLAAREGSYEAAKILLDHFANRDITDHMDRLPRDVARDRM
HHDIVRLLDEYNVTPSPPGTVLTSALSPVICGPNRSFLSLKHTPMGKKSRRPSAKSTMPTSLPNLA
KEAKDAKGSRRKKSLSEKVQLSESSVTLSPVDSLESPHTYVSDTTSSPMITSPGILQASPNPMLAT
AAPPAPVHAQHALSFSNLHEMQPLAHGASTVLPSVSQLLSHHHIVSPGSGSAGSLSRLHPVPVPA
DWMNRMEVNETQYNEMFGMVLAPAEGTHPGIAPQSRPPEGKHITTPREPLPPIVTFQLIPKGSIAQ
PAGAPQPQSTCPPAVAGPLPTMYQIPEMARLPSVAFPTAMMPQQDGQVAQTILPAYHPFPASVGK
YPTPPSQHSYASSNAAERTPSHSGHLQGEHPYLTPSPESPDQWSSSSPHSASDWSDVTTSPTPG
GAGGGQRGPGTHMSEPPHNNMQVYA

NICD2RA amino acid sequence:

SYPLVSVVSESLTPERTQLLYLLAVAVVIILFIILLGVIMAKRKRKHGSLWLPEGFTLRRDASNHKRRE
PVGQDAVGLKNLSVQVSEANLIGTGTSEHWVDDEGPQPKKVKAEDEALLSEEDDPIDRRPWTQQ
HLEAADIRRTPSLALTPPQAEQEVDVLDVNVRGPDGCTPLMLASLRGGSSDLSDEDEDAEDSSAN
IITDLVYQGASLQAQTDRTGEMALHLAARYSRADAAKRLLDAGADANAQDNMGRCPLHAAVAADA
QGVFQILIRNAVTDLDARMNDGTTPLILAARLAVEGMVAELINCQADVNAVDDHGKSALHWAAAVN
NVEATLLLLKNGANRDMQDNKEETPLFLAAREGSYEAAKILLDHFANRDITDHMDRLPRDVARDRM
HHDIVRLLDEYNVTPSPPGTVLTSALSPVICGPNRSFLSLKHTPMGKKSRRPSAKSTMPTSLPNLA
KEAKDAKGSRRKKSLSEKVQLSESSVTLSPVDSLESPHTYVSDTTSSPMITSPGILQASPNPMLAT
AAPPAPVHAQHALSFSNLHEMQPLAHGASTVLPSVSQLLSHHHIVSPGSGSAGSLSRLHPVPVPA
DWMNRMEVNETQYNEMFGMVLAPAEGTHPGIAPQSRPPEGKHITTPREPLPPIVTFQLIPKGSIAQ
PAGAPQPQSTCPPAVAGPLPTMYQIPEMARLPSVAFPTAMMPQQDGQVAQTILPAYHPFPASVGK
YPTPPSQHSYASSNAAERTPSHSGHLQGEHPYLTPSPESPDQWSSSSPHSASDWSDVTTSPTPG
GAGGGQRGPGTHMSEPPHNNMQVYA

S6. Amino acid sequence of NICD2 and NICD2RA.
The amino acid sequence of truncated human NICD2 and NICD2RA. The transmembrane domain is boxed in red dashed line. ARG(R1934) and ALA(A1934) are highlighted. Red arrow marks gamma secretase cleavage sites that will generate stable NICD2s.
