# Supplementary material for: Loss of Notch dimerization perturbs intestinal homeostasis by a mechanism involving HDAC activity
Source: PLoS Genet. 2024 Dec 12;20(12):e1011486. doi: 10.1371/journal.pgen.1011486 (PMC11670933; doi:10.1371/journal.pgen.1011486)
Supplement: S2 Data — (DOCX) [file pgen.1011486.s008.docx]

**DIA Quantification Proteomics Report**, Modified from Beijing Genome Institute summary report 400-706-6615. Technical Support E-mail: [info@bgitechsolutions.com](mailto:info@bgitechsolutions.com) Website: [www.bgitechsolutions.com](http://www.bgitechsolutions.com)

#
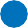
Results

## Project Overview and Quality Control Analysis

- 1. Overview

In this project, Q-Exactive HF X (Thermo Fisher Scientific, San Jose, CA) was used to acquire mass spectrometry (MS) data for 6 samples in Data Independent Acquisition (DIA) mode, 50367 peptide and 3580 protein were quantitated. Quantification of peptides and proteins was performed using MSstats software packages. The quantitative statistics of each sample are as follows:

Table 1 Overview of quantitative results for each sample

| **Name** | **Peptide number** | **Protein number** |
| --- | --- | --- |
| HT_3 | 23763 | 2821 |
| HT_6 | 12859 | 1946 |
| N2_1 | 34043 | 3355 |
| N2_4 | 26602 | 3192 |
| NR_2 | 26147 | 3225 |
| NR_5 | 22733 | 3072 |

- 1. Quality control

The quality of the DIA data was evaluated based on intra-group coefficient of variation (CV), principal component analysis (PCA), and quantitative correlation of samples. When the sample size is large, quality control (QC) samples, which are generally a mixture of all samples, are inserted intermittently between the continuous original samples. Thus, experimental conditions can be evaluated by the following QC analysis to ensure stability and repeatability of the experiment.


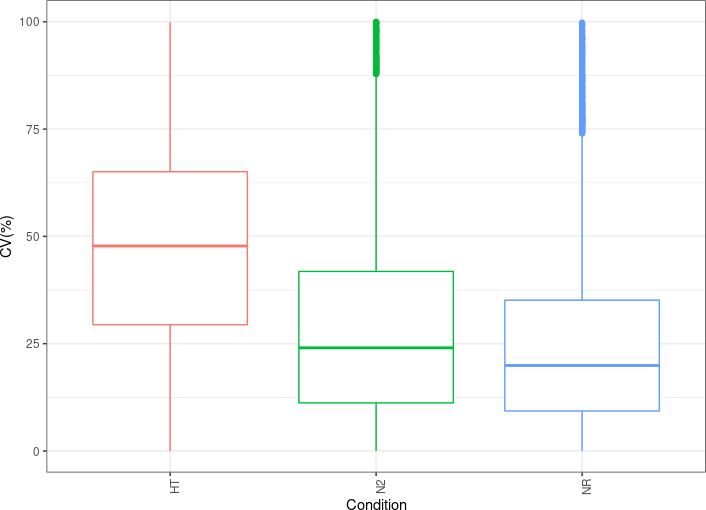


Figure 1 CV distribution.

This analysis was used to calculate intra-group CV of diﬀerent sample groups. The X-axis denotes the sample group and the Y-axis denotes the corresponding CV. (When the sample size in project is large, there is a QC group to be used to evaluate stability and repeatability of the experiment).

In this project, Pearson correlation coefficient of all protein expression between every two samples was calculated to demonstrate correlation of protein quantification between samples and was represented as a heat map as follows:


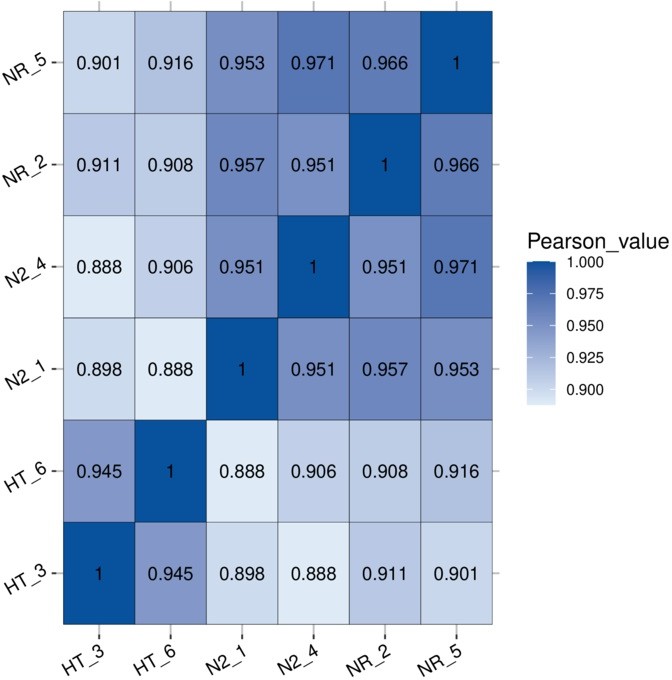


Figure 2 Heat map of sample correlation analysis.

Both X and Y axes represent samples. The color represents the correlation coefficient (the deeper color represents the higher correlation; the lighter color represents the lower correlation).

## Basic statistical chart of the protein identification results

The following figures are the basic statistics of the protein identification results, which are unique peptide distribution, protein mass distribution and protein coverage distribution respectively.
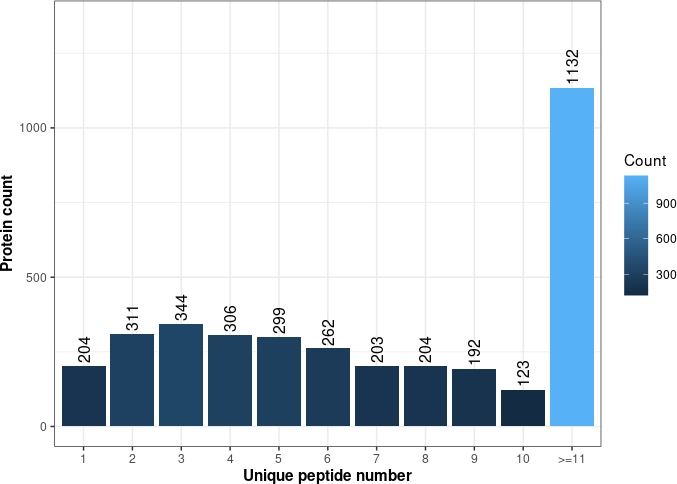


Figure 3 Unique peptide distribution.

The X-axis is the number of unique peptides for each protein, and the Y-axis is the number of proteins.


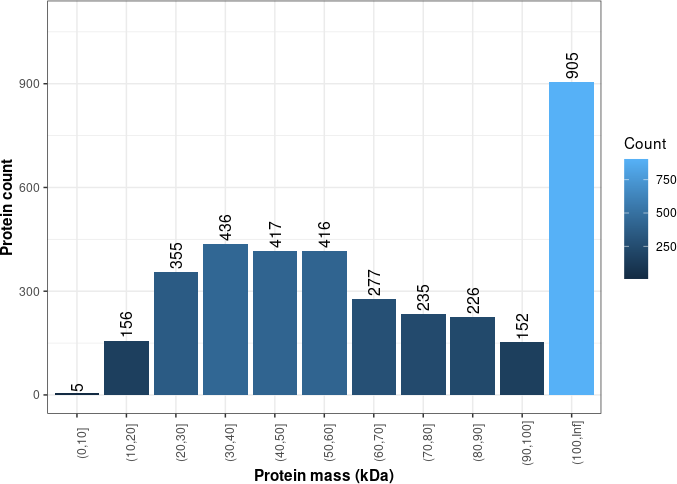


Figure 4 Protein mass distribution.

The X-axis is protein mass interval (Kilodalton), and the Y-axis is the number of proteins.


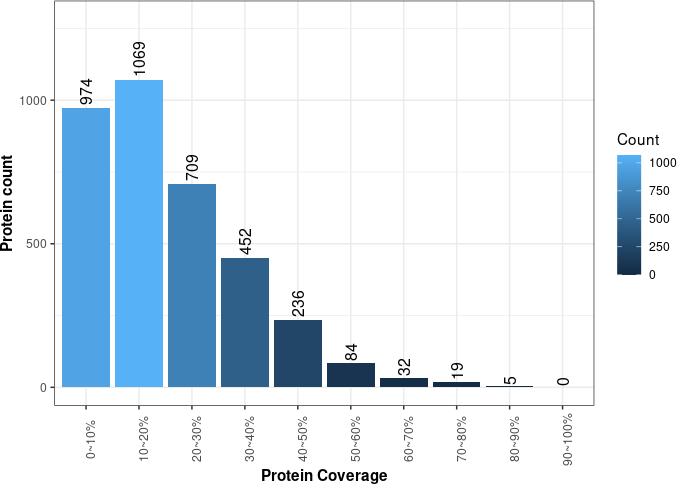


Figure 5 Protein coverage distribution.

The X-axis is coverage percentage interval, and the Y-axis is the number of proteins.

## Protein Quantification

3.1 Principal component analysis

Principal component analysis (PCA) is a method of dimension reduction that combines multiple variables to a new set of integrated variables, and then selects several (usually 2-3) to represent as much original information as possible, thus to achieve the purpose of dimension reduction. PCA is mainly used to observe the trend of separation between groups in the experimental model, and whether there are exceptional value points, and reflect the inter- and intra- group variations from the original data.


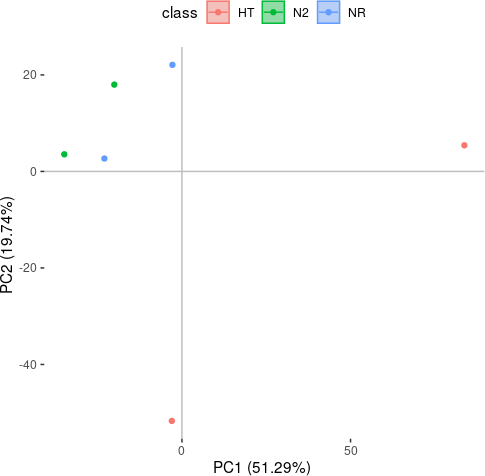


Figure 6 Principal component analysis.

The X-axis is the first principal component and the Y-axis is the second principal component.

#
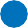
 Methods

## Pipeline Introduction

This project was analyzed using next generation label-free quantitative proteomics technology. In data independent acquisition (DIA) mode, it can deliver unprecedented proteomic coverage while enabling accurate and highly repeatable quantification for large amounts of proteins per sample. The DIA analysis pipeline provides an ideal diﬀerential proteomic analysis or a proteomic quantification platform for large amounts of samples.

The DIA analysis pipeline is based on three essential steps:

1. Spectral library construction: A spectral library collects all detectable non-redundant, high- quality peptide information (MS/MS spectra) of the sample that can be used as a peptide identification template for subsequent data analysis. It contains fragment ion intensity and retention time that characterize the peptide spectrum. The spectral library is constructed from samples of interest by using data dependent acquisition (DDA) technique.
2. Large sample data acquisition in DIA mode: Data independent acquisition (DIA, also called SWATH) mode utilizes the latest high-resolution mass spectrometer to simultaneously acquire peptide ion characteristics in mass and retention time space. Compared to traditional technique of extracting single ion for fragmentation analysis, in DIA mode the mass spectrometer is set to a wide precursor ion window to collect product ions in turn. Thus complete collection of all detectable protein peak information in the sample and high-reproducible analysis of large number of samples is achieved.
3. Data analysis: Identificaton and quantification of peptides and proteins were obtained from DDA spectral library by deconvolution of the DIA data. MSstats software package was used to perform diﬀerential analysis, followed by functional analysis of the diﬀerential proteins.

## Experimental Pipeline

- 1. Protein extraction
  2. Protein extraction quality control

(1) Bradford quantification

Standard proteins (0.2μg/μL BSA) 0, 2, 4, 6, 8, 10, 12, 14, 16, 18μL were sequentially added to the 96-well microtiter plates A1 to A10, followed by the addition of pure water 20, 18, 16, 14, 12, 10, 8, 6, 4, 2μL, and then 180μL of Coomassie Brilliant Blue G-250 Quantitative Working Solution was added to each well. The OD595 was measured with a microplate reader, and a linear standard curve was drawn based on the OD595 and protein concentration. Diluted the protein solution to be tested several times, added 180μL of the quantitative working solution to 20μL of the protein solution, and read at OD595. The sample protein concentration was calculated from the standard curve and sample OD595.

(2) SDS-PAGE

Each 10μg of protein solution was mixed with an appropriate amount of loading buﬀer, heated at 95°C for 5 minutes, centrifuged at 25,000g for 5 minutes, and the supernatant was loaded into a well of a 12% SDS polyacrylamide gel. 120V constant pressure electrophoresis for 120 minutes;

After electrophoresis, Coomassie blue staining was carried out for 2 hours, after which an appropriate amount of decolorizing solution (40% ethanol 10% acetic acid) was added to the shaker to decolorize for 3 to 5 times for 30 minutes each time.

- 1. Protein enzymatic hydrolysis

1. Take 100μg of protein solution per sample and dilute with 50mM NH4HCO3 by 4 times volumes;
2. Add 2.5μg of Trypsin enzyme in the ratio of protein: enzyme = 40:1, and digest for 4 hours at 37°C;
3. Enzymatic peptides were desalted using a Strata X column and vacuumed to dryness.
   1. High pH RP separation

Equal amount of peptides were extracted from all samples to mix, and the mixture was diluted with mobile phase A (5% ACN pH 9.8) and injected. The Shimadzu LC-20AB HPLC system coupled with a Gemini high pH C18 column (5μm, 4.6 x 250mm) was used. The sample was subjected to the column and then eluted at a flow rate of 1mL/min by gradient: 5% mobile phase B (95% ACN, pH 9.8) for 10 minutes, 5% to 35% mobile phase B for 40 minutes, 35% to 95% mobile phase B for 1 minute, flow Phase B lasted 3 minutes and 5% mobile phase B equilibrated for 10 minutes. The elution peak was monitored at a wavelength of 214nm and component was collected every minute. Components were combined into a total of 10 fractions, which were then freeze-dried.

- 1. DDA and DIA analysis by nano-LC-MS/MS

The dried peptide samples were reconstituted with mobile phase A (2% ACN, 0.1% FA), centrifuge at 20,000g for 10 minutes,and the supernatant was taken for injection. Separation was carried out by a Thermo UltiMate 3000 UHPLC liquid chromatograph. The sample was first enriched in the trap column and desalted, and then entered a tandem self-packed C18 column (150μm internal diameter, 1.8μm column size, 35cm column length), and separated at a flow rate of 500nL/min by the following eﬀective gradient: 0~5 minutes, 5% mobile phase B (98% ACN, 0.1% FA); 5~120 minutes, mobile phase B linearly increased from 5% to 25%; 120~160 minutes, mobile phase B rose from 25% to 35%; 160~170 minutes, mobile phase B rose from 35% to 80%; 170~175 minutes, 80% mobile phase B; 175~180 minutes, 5% mobile phase B. The nanoliter liquid phase separation end was directly connected to the mass spectrometer as the following settings.

For DDA analysis, LC separated peptides were ionized by nanoESI and injected to tandem mass spectrometer Q-Exactive HF X (Thermo Fisher Scientific, San Jose, CA) with DDA (data-dependent acquisiton) detection mode. The main settings were: ion source voltage 1.9kV; MS scan range 350~1,500m/z; MS resolution 120,000, maximal injection time (MIT) 100ms; MS/MS collision type HCD, collision energy NCE 28; MS/MS resolution 30,000, MIT 100ms, dynamic exclusion duration 30 seconds. The start m/z for MS/MS was fixed to 100. Precursor for MS/MS scan satisfied: charge range 2+ to 6+, top 20 precursors with intensity over 5E4. AGC was: MS 3E6, MS/MS 1E5.

**3 Bioinformatic Analysis Pipeline**

For DIA analysis, LC separated peptides were ionized by nanoESI and injected to tandem mass spectrometer Q-Exactive HF X (Thermo Fisher Scientific, San Jose, CA) with DIA (data-independent acquisiton) detection mode. The main settings were: ion source voltage 1.9kV; MS scan range 400~1,250m/z; MS resolution 120,000, MIT 50ms; 400~1,250m/z was eqaully divided to 45 continuous windows MS/MS scan. MS/MS collision type HCD, MIT was auto mode. Fragment ions were scanned in Orbitrap, MS/MS resolution 30,000, collision energy was distributed mode: 22.5, 25, 27.5, AGC was 1E6.

3.1 Database selection

The selection of database is an important step in MS based protein identification, and the final identified protein sequences are from the selected database.

Currently databases in use can be divided into three main categories:

1) UniProt protein database

UniProt is the most informative and resourceful protein database. It consists of data from three major databases, i.e. Swiss-Prot, TrEMBL and PIR-PSD. It is a data set verified by experts and consists of two parts: UniProtKB/Swiss-Prot (with reviewed, manually annotated entries) and UniProtKB/TrEMBL (with unreviewed, automatically annotated entries). In general, it is recommended to give priority using the subset of UniProtKB/Swiss-Prot for protein identification. When it aims to find novel sequences (such as alternative splicing, new transcripts) or to identify allied species, UniProtKB/TrEMBLdatatbasecanbeconsidered.

2) The protein databases based on genome annotation

The databaseses mainly include a series of databases derived from NCBI and Ensembl gene annotation databases. Among them, we choose protein database from reference sequence (RefSeq) of NCBI, which is a non-redundant proteome database. It is widely used in the analysis of multi-omics studies due to the importance of the NCBI annotation system. NCBI's RefSeq provides reference sequence for molecules that are naturally involved in central dogma, from chromosomes to mRNA and proteins. The RefSeq standard provides a basis for functional annotation of the human genome. It provides a stable reference for mutation analysis, gene expression studies, and polymorphic discovery. In addition, NCBI provides completed non-redundant protein sequence database (NCBI_nr), including animal, plant, microbial, bacterial and other taxonomy. Since the database is derived from various sources (including GeneBank, RefSeq, SwissProt, PDB, etc.), unless the species is without complete genome annotation, or it is necessary to search for homologous, it is not recommended to use this database for protein identification.

Ensembl aims to develop a software package with automatic annotation and maintenance for the eukaryotic genome. Ensembl has relatively complete and consistent genomic, transcriptome, and proteomic annotation information, which is ideal for multi omics analysis.

3) Databases from other sources

They usually refer to the protein target databases provided by the client, or new gene sequence generated from genome or transcriptome sequencing de novo assembly. They may also contain sequences of new features such as alternative splicing, mutation site, fusion genes, etc.

3.2 DIA data analysis

The DIA data was analyzed using the iRT peptides for retention time calibration. Then, based on the target-decoy model applicable to SWATH-MS, false positive control was performed with FDR 1%, therefore obtaining significant quantitative results.

3.3 MSstats differential analysis

MSstats [*Choi, Meena, et al. MSstats: an R package for statistical analysis of quantitative mass spectrometry- based proteomic experiments. Bioinformatics 30.17 (2014): 2524-2526*] is an R package from the Bioconductor repository. It can be used for statistical evaluation of significant differences in proteins or peptides from different samples, and is widely used in targeted proteomics MRM, label free quantitation, and SWATH quantitative experiments. The core algorithm is linear mixed effect model. The process preprocessed the data according to the predefined comparison group, and then performed the significance test based on the model. Thereafter, differential protein screening was performed based on the fold change >2 and Pvalue<0.05 as the criterion for the significant difference. At the same time, the enrichment analysis is performed on the differential proteins.
